# Relative age effect and second-tiers: No second chance for later-born players

**DOI:** 10.1371/journal.pone.0201795

**Published:** 2018-08-08

**Authors:** Ante Rađa, Johnny Padulo, Igor Jelaska, Luca Paolo Ardigò, Luca Fumarco

**Affiliations:** 1 University of Split, Faculty of kinesiology, Split, Croatia; 2 University eCampus, Novedrate, Italy; 3 Tunisian Research Laboratory Sports Performance Optimization, National Center of Medicine and Science in Sport, Tunis, Tunisia; 4 School of Exercise and Sport Science, Department of Neurosciences, Biomedicine and Movement Sciences, University of Verona, Verona, Italy; 5 STATEC Research, Luxembourg, Grand Duchy of Luxembourg; University of North Carolina at Chapel Hill, UNITED STATES

## Abstract

The main objective of this research was to determine the existence of relative age effect (RAE) in five European soccer leagues and their second-tier competitions. Even though RAE is a well-known phenomenon in professional sports environments it seems that the effect does not decline over the years. Moreover, additional information is required, especially when taking into account second-tier leagues. Birthdates from 1,332 first-tier domestic players from France, England, Spain, Germany and Italy and birthdates from 1,992 second-tier domestic players for the 2014/2015 season were taken for statistical analysis. In addition to standard statistical tests, the data were analyzed using econometric techniques for count data using Poisson and negative binomial regressions. The results obtained confirmed a biased distribution of birthdates in favor of players born earlier in the calendar year. For all of the five first-tier soccer leagues there was an unequal distribution of birthdates (France *χ*^2^ = 40.976, *P*<0.001; England *χ*^2^ = 21.892, *P* = 0.025; Spain *χ*^2^ = 24.690, *P* = 0.010; Germany *χ*^2^ = 22.889, *P* = 0.018; Italy *χ*^2^ = 28.583, *P* = 0.003). The results for second-tier leagues were similar (France *χ*^2^ = 46.741, *P*<0.001; England *χ*^2^ = 27.301, *P* = 0.004; Spain *χ*^2^ = 49.745, *P*<0.001; Germany *χ*^2^ = 30.633, *P* = 0.001; Italy *χ*^2^ = 36.973, *P*<0.001). Econometric techniques achieved similar results: estimated effect of month of birth, i.e., long-term RAE on players’ representativeness, is negative (statistically significant at the 1% level). On average, one month closer to the end of the year reduces the logs of expected counts of players by 6.9%. Assuming this effect as linear, being born in the month immediately before the cut-off date (i.e., December/August), reduces the logs of expected counts of players by approximately 75.9%. Further, I_D_ (index of discrimination, that is, the ratio between the expected counts of players born in the middle of the first and the twelfth month of the selection year) is 2.13 and 2.22 for the first- and second-tier, respectively. In other words, in the top five European first-tier and second-tier leagues, one should expect the number of players born in the first month of the calendar year to be twice the number of those born in the last month. The RAE in the second-tiers is the same as in the first-tiers, so it appears that there is no second chance for later born players. This reduces the chances to recover talented players discarded in youth simply because of lower maturity.

## Introduction

The Relative Age Effect (RAE) is a very well-known phenomenon in the sports literature. Of what does it consist? RAE is a bias descriptor, mainly used to refer to youth sports levels; when this bias is present, according to a normalized distribution of live births there are more participants born earlier than expected in a relevant selection period (and, vice versa, fewer participants born later [1]). Maturity differences in childhood sports competitions result in differences in performance between children. Reasonably enough, one may think that these performance gaps should be short-lived as the importance of maturity differences decreases with age and, thus, should not be reflected in a gap in adulthood. However, these maturity-induced performance gaps are easily mistaken for sheer talent differences and, because of developmental constraints [1] and social, as well as psychological factors [2], these differences may be reflected later on in differences in terms of representativeness in professional sports, that is, those athletes who were the oldest in their youth group-age (i.e., relatively old players) are over-represented.

The RAE is present in a wide array of sports and soccer is no exception. This phenomenon was first studied in soccer in the early 1990s when Barnsley et al. [3] analyzed the presence of RAE in the youth 1990 FIFA World Cup. Since then, many studies have confirmed the RAE phenomenon in various youth and professional soccer competitions, both at the international level [4–6] and the national level [5,7–13].

Is there an RAE in lower-level professional competitions, such as second-tier national leagues? This is a relevant question because one may think of these lower leagues as back-doors to first-tier competitions and, thus, they are especially important to those players who initially struggled the most to emerge, that is, the players who were the youngest in their youth age-group (i.e., relatively young players). However, to the best of our knowledge, no study has so far assessed the presence of the RAE in second-tier competitions. We contribute to the literature by filling this gap.

Moreover, this paper contributes to the dissemination of recent methodological advances in the RAE literature. In addition to standard statistical tests, such as Chi-square tests, we investigate the data using econometric techniques for count data. This is only the third study to do so—the first was that of Doyle and Bottomley [4] and the second was that of Brustio et al. [14]. The advantage of these techniques is that they help us to understand the magnitude of the phenomenon and enable responses to questions such as: (i) how much less represented are late-born players and (ii) how much less represented are these players in second-tiers compared to first-tiers.

In our analyses, we investigate first- and second-tier data from the 2014/2015 season from Spain, Germany, Italy, England and France—perhaps the five most important European leagues. We investigate only domestic players because we pay particular attention to a commonly neglected aspect: different countries may adopt different cut-off dates (i.e., the date set by soccer federations to establish how to divide players into different age groups) and may enforce them to a different degree (i.e., it might be possible to circumvent formal youth age-grouping rules for several reasons; see Williams [6] for an example). In the absence of information about cut-off dates, researchers often assume uniformity of cut-off dates and this may be problematic and lead to spurious evidence of RAE [15].

The remainder of this paper proceeds as follows. The materials and methods section discusses the data and illustrates the Poisson and negative binomial methods. The results section presents the principal findings, whereas the discussion and conclusions draw out the implications for theory and practice.

## Materials and methods

### Participants

We analyzed players from first- and second-tier soccer leagues in France, Germany, Italy, England and Spain during the 2014/2015 season [16]. Foreign players within each league were not analyzed to ensure that the applied cut-off date is the correct one. For the two English leagues (i.e., Premier League and Championship), British players (i.e., from England, Scotland, Wales and Northern Ireland) are treated as if they had the same citizenship. Our sample can be divided into two subsamples: the first subsample consists of N_1_ = 1,332 first-tier domestic players, whereas the second subsample consists of N_2_ = 1,992 second-tier domestic players. For the period of analysis, France, Germany, Italy and Spain applied the same cut-off date, that is, January 1, whereas England applied a different cut-off date, September 1. To account for this difference, we have rescaled the English calendar so that the first month of the calendar year is September, whereas the last month is August. All experimental procedures were approved by the institutional human ethics committee, which followed ethical standards (University of Split, Human Research Ethics Committee).

### Procedures

#### Chi-square test

The Chi-square (*χ*^2^) test was used to investigate the differences between observed and expected frequencies of birthdates. The expected frequencies are computed assuming a uniform distribution of dates of birth within the year. As a measure of the effect size for the *χ*^2^ applied to a 1×12 contingency table, the W coefficient was calculated:

$$W=\sqrt{\frac{\chi^{2}}{n}}$$

*W*≤0.1 was considered to be a small effect, W ∈ (0.1,0.3) was considered a medium effect and *W*>0.3 was considered a large effect [17].

We are aware that for each country it would be more appropriate to compute the expected frequencies based on the parent birthdates distribution of the country’s population [18]. However, it turned out that retrieving this data for all of the five countries in a consistent manner was not feasible.

The *χ*^2^ test was performed separately for the first- and second-tier subsamples. Moreover, we performed this test for the overall sample and for France, Germany, Italy, England and Spain separately.

For this test, and in line with the sports sciences tradition of reporting results from parametric tests, *P*<0.05 was used as a threshold for statistical significance.

#### Poisson and negative binomial regressions

There are a few alternative count data econometric techniques. The most important of these are perhaps the Poisson and the negative binomial regression [19]. The choice between the two is data-driven. As is well established by the RAE literature, in the presence of the RAE one should expect the frequency of players’ dates of birth in a given tournament to be right-skewed, that is, the left tail is fatter than the right tail. A possible implication is that the variance in the frequency of the monthly birthdates could be larger than its mean; in other words, the outcome variable is *overdispersed* [19]. The consequence is that the negative binomial regression method could be preferred over the Poisson regression since the latter assumes that the variance equals the mean. In turn, when we account for overdispersion there is more uncertainty in our estimates. As a consequence, the confidence intervals tend to be larger, there are greater chances that they include zero and, thus, there are fewer chances to reject null hypotheses that would not be rejected using the Poisson regression. In the following analyses, we use the Poisson regression as the main instrument of analysis, whereas the negative binomial regression is used as a robustness check.

In both the Poisson and the negative binomial regressions, the frequency count of an outcome variable is explained by the given independent variables. Both regression methods can be illustrated in the simplified model (1):

$$Fr\_y_{m}=e^{\left(\beta_{0}+\beta_{1}Month_{m}+\gamma Country_{m}+\lambda Sec.League_{m}+\omega Sec.League_{m}\times Month_{m}\right)}$$ (1)

where *Fr_y*_*m*_ represents the players’ counts *per* month of birth and *m*. *Month*_*m*_ is the month of birth, which goes from 1 to 12 (1 is the month that starts with the cut-off date, whereas 12 is the month immediately before that date). In our dataset, most of the countries share the same cut-off date, that is, January 1 and, thus, 1 equals January—this is the reference month, whereas 12 equals December. The only exception is England in which age groups are formed with children born from September 1 (month 1) of year t to August 31 of year t+1 (month 12). The months of birth for English players are, thus, rescaled accordingly. Note that since the right-hand side is exponentiated—according to the nature of count regression methods—the estimated coefficients have to be interpreted in terms of the percentage change of log counts. For instance, for a one-unit increase in the variable Month, the expected log count of the number of players born in that month increases by ${\hat{\beta }}_{1}\%$. The estimate of *β*_1_, that is, ${\hat{\beta }}_{1}$, is our *primary focus* because it provides the magnitude of the RAE. *Country*_*m*_ is a vector, which is composed of dummy variables for the countries we are studying (i.e., France, Germany, Italy or Spain, and England is the reference country); *γ* is the vector of coefficients associated to them. This is how we interpret their coefficients. For instance, $\hat{\gamma }$, associated with the dummy for France, communicates the effect on the log counts of being in France, as compared to England. *Sec*.*League*_*m*_ is a dummy variable that equals 1 if we are observing a second-tier league rather than a first-tier league. *Sec*.*League*_*m*_*×Month*_*m*_ is the interaction between month and league and $\hat{\omega }$ is the *secondary focus* of our analysis because it tells us whether the RAE in second-tier leagues differs from that in first-tier leagues. The results include the estimate of the *alpha parameter*, which accounts for overdispersion—this parameter is not shown in Equation (1).

The analyses are carried out using the statistical software, Stata v15. We use the command “poisson” for the Poisson regression and the command “nbreg” for the negative binomial regression. For illustrative purposes, examples are available at https://stats.idre.ucla.edu/stata/dae/negative-binomial-regression/ and at https://stats.idre.ucla.edu/stata/dae/poisson-regression/.

Note that there are alternative regression models that account for the overdispersion issue, such as the quasi-Poisson regression. However, we focus on just one model and we do this for at least two reasons: (i) we would like to focus on one new method at a time to keep the study clear and short and (ii) considering that a large portion of the readers may not be acquainted with Stata, one should consider the relevant advantage of the output provided by such statistical software from the negative binomial regression, which automatically includes results from the test for overdispersion.

The Stata syntax for the tables in this paper is available in the online material (S1 and S2 Files).

For these econometric analyses, and in line with Doyle and Bottomley [4] with regard to reporting results from non-parametric analyses, such as econometric analyses, we used multiple thresholds for the statistical significance and these are reported underneath each table.

## Results

Our analyses are conducted with *χ*^2^ tests and two count data econometric techniques: the Poisson regression as well as negative binomial regression.

### Chi-square results

**Fig 1:**
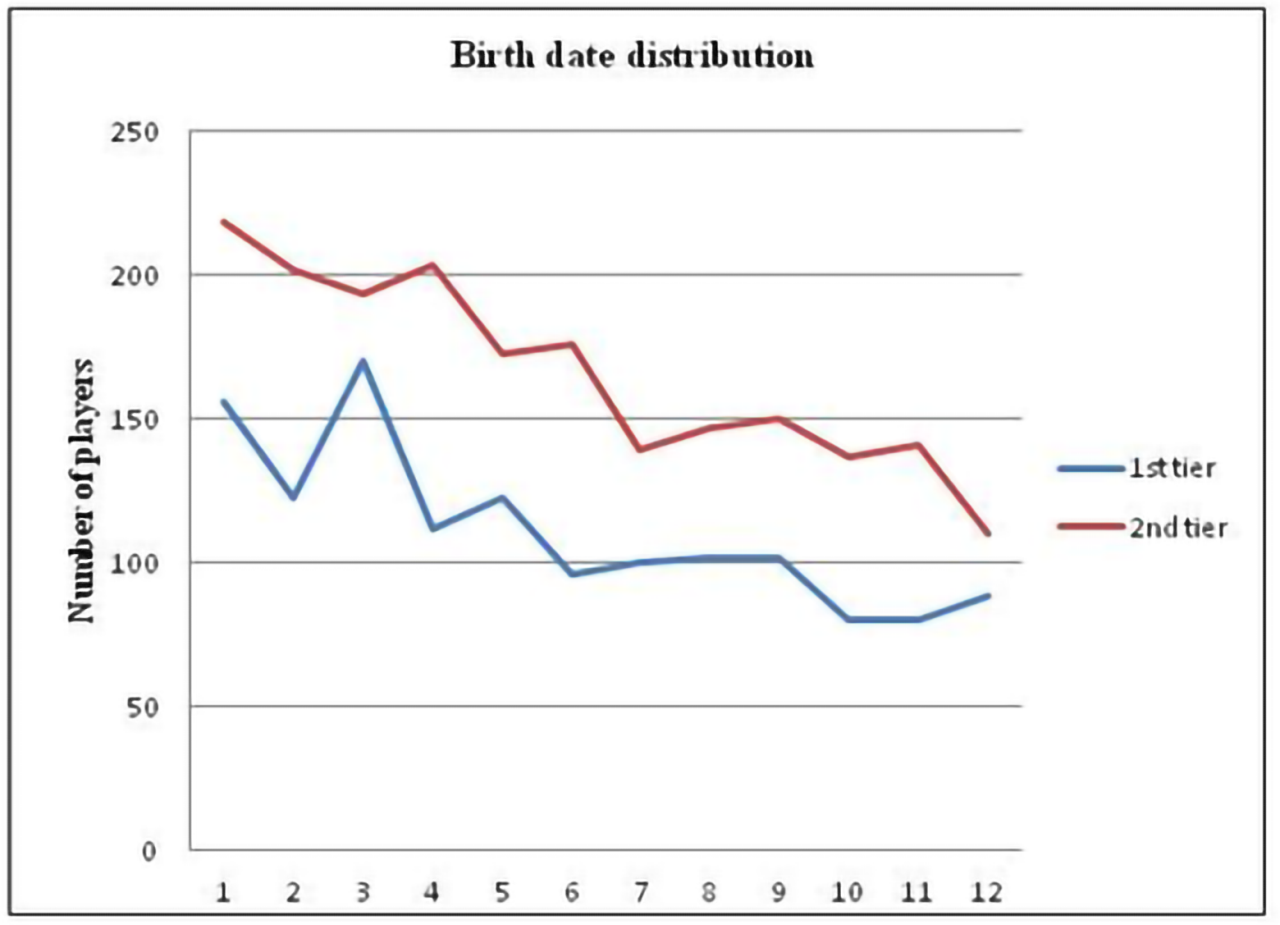
Soccer players’ birth dates monthly distributions for first- and second-tiers of the top five European leagues.

Fig 1 and Table 1 illustrate the counts of first- and second-tier players for the overall sample and for each country separately. In addition, the table reports the *χ*^2^ test results. Significant differences between counts within months were observed consistently for both first-tiers and second-tiers, for all leagues as well as for overall data.

**Table 1:** Counts and *χ*^2^ test of first- and second-tier players for the overall sample and for each country separately.

	Overall		Germany		France		Italy		England		Spain	
	First-tier	Second-tier	First-tier	Second-tier	First-tier	Second-tier	First-tier	Second-tier	First-tier	Second-tier	First-tier	Second-tier
	N	N	N	N	N	N	N	N	N	N	N	N
**Months**												
1	156	219	34	38	21	42	34	33	30	47	37	59
2	123	202	21	25	28	41	29	37	16	40	29	59
3	170	194	23	31	40	33	35	51	31	30	41	49
4	112	204	16	38	24	42	22	50	18	24	32	50
5	123	173	17	21	20	18	33	56	19	28	34	50
6	96	176	13	32	14	34	31	39	12	25	26	46
7	100	139	15	17	21	25	21	34	13	26	30	37
8	102	147	17	31	31	16	18	41	15	28	21	31
9	102	150	19	29	15	21	20	31	24	46	24	23
10	80	137	14	21	10	18	14	27	24	41	18	30
11	80	141	10	24	13	19	15	27	27	48	15	23
12	88	110	17	9	13	15	16	18	20	42	22	26
N	1,332	1,992	216	316	250	324	288	444	249	425	329	483
*χ*^2^(11)	78.865	74.879	22.889	30.633	40.976	46.741	28.583	36.973	21.892	27.301	24.690	49.745
*P*-value	<0.001	<0.001	0.018	0.001	<0.001	<0.001	0.003	<0.001	0.025	0.004	0.010	<0.001
W	0.243	0.194	0.326	0.311	0.405	0.380	0.298	0.289	0.296	0.253	0.274	0.321

### Poisson and negative binomial regressions

Table 2 illustrates the results from the negative binomial and Poisson regression models. Note that for both analyses, we investigate 120 observations, that is, one observation *per* month (12), *per* country (5) and *per* tier (2).

**Table 2:** RAE on frequency of month of birth in the first- and second-tiers of the five most important European leagues.

	Models	
	Poisson	Negative binomial
Variables		
France^a^	-0.156***	-0.155**
	(0.057)	(0.062)^b^
Germany	-0.232***	-0.230***
	(0.058)	(0.063)
Italy	0.087	0.089
	(0.053)	(0.059)
Spain	0.191***	0.190***
	(0.052)	(0.058)
Month	-0.069***	-0.069***
	(0.008)	(0.009)
Sec.League	0.423***	0.424***
	(0.069)	(0.077)
Sec.League*Month	-0.004	-0.004
	(0.010)	(0.012)
N	120	120
Pseudo *R*-squared	0.350	0.175
Alpha	0.007	
*P*-value, H_0_: Alpha = 0	0.065^c^	

*** *P*<0.01

** *P*<0.05

* *P*<0.1

^a^ England is the reference country.

^b^ Standard error in parenthesis.

^c^ *P*-value from the likelihood-ratio test on the equality of the variances between in the two models.

These results reveal five interesting insights. First, the estimated effect of month of birth—which proxies the estimated long-term RAE on players representativeness in the first- and second-tiers of the five most important European leagues—is negative and statistically significant at the 1% level in both models. The estimated effect of *Month* tells us that, holding the country and tier as fixed, one month closer to the end of the year, on average, reduces the logs of expected counts of players by 6.9%—this is true whether we refer to the estimates of the Poisson or the negative binomial regression. Assuming that this effect is linear, we can compute the Index of Discrimination (I_D_ [4,20]), that is, *e*^Month*N^ with N being the number of months between two players’ birthdays. In other words, this index provides the ratio of the expected number of players born in the first month to the same statistic for those players born in the last month. Given that we are using information on month of birth—rather than day of birth—this index can be computed in at least two ways: by referring to the middle of the months or to their extremities. First, consider players born in the middle of the month immediately before the cut-off date (i.e., December or August in England); for every player selected from the middle of month 1, only 0.468 (= *e*^–0.759^; where 0.759 = 6.9*11) players would be selected from the middle of month 12. Second, consider players born at the extremities (i.e., January 1 versus December 31 or September 1 versus August 31); for every player selected from the beginning of month 1, only 0.437 (= *e*^–0.828^; where 0.828 = 6.9*12) players would be selected from the end of month 12.

Second, teams have different sizes in different countries. In France and Germany the logs of the expected counts of players are lower—about 15% and about 23%, respectively—than that of the reference country in our analyses, England. In Spain, the log of the expected counts of players is about 19% higher than that of England, whereas Italy does not differ from England in this respect. These estimates capture the combined effect of several factors that are country-specific and that may affect the number of players born in a single month in a given country and tier (e.g., the number of foreign players per team) and, thus, we refrain from providing their precise interpretation (some disciplines do not report at all results that refer to these so-called “fixed-effects”). However, for educational purposes, we would like the reader to appreciate the utility of the exponential form embodied in count regression models: the estimated country-specific coefficients approximate the percentage difference in the number of domestic players in each team of the first two leagues of a given country compared to England. For instance, the coefficient of France is -0.156, which tells us that in our sample, there are 15.6% fewer French footballers than English footballers.

Third, there are more players in the second-tiers. On average, in second-tier leagues, the logs of expected counts of players are about 42% larger than in the first-tier leagues. This should not be surprising since, in general, there are more teams in second-tier leagues.

Fourth, the RAE in second-tiers does not differ from that in first tiers. The effect of the interaction term “*Sec*.*League×Month*” is not statistically significant at any conventional level. This is the most important result of our paper because it provides evidence that the RAE does not vary by tier.

Fifth and final, the table reports the result from the test on the significance of the overdispersion parameter, that is, Alpha. This test does not reject the null hypothesis at the 10% significance level, which implies that for this sample, the negative binomial model would be more appropriate than the Poisson model. However, neither the magnitude nor the statistical significance of our main results (i.e., the estimated effects of Month and of its interaction with Sec.League) appears to be impacted by the choice between the Poisson and the negative binomial regression. Therefore, the following tables report results from the Poisson regression alone—results from the negative binomial regression can be provided upon request.

As a robustness check, we repeat this analysis with both the Poisson and the negative binomial regressions, first, only on domestic players with no second citizenship and then only on players born from year 1990 on. These two subsamples are more likely to contain players that actually trained under the same cut-off date in youth (e.g., in the mid-1990s Germany changed the cut-off date from August to January 1 and, therefore, German players born at the end of the 1980s might have been subject to the August cut-off). Both analyses report results that are similar to the main analysis. The results are omitted for brevity but can be provided upon request.

Table 3 provides the results by country. For sake of brevity, we only report results from the Poisson regression. This decision does not affect the estimates: the test on the significance of the overdispersion parameter rejects the null hypothesis (i.e., *P*-value>0.1) only for France and the negative binomial regression for that country does not return different coefficients. Note that, since results for the different countries are reported separately in each column, the results are obtained from analyzing 24 observations, that is, one observation *per* month *per* tier.

**Table 3:** RAE on frequency of month of birth in the first- and second-tiers by country.

	Country				
	England	France	Germany	Italy	Spain
Variables					
Month	-0.056***	-0.074***	-0.065***	-0.079***	-0.068***
	(0.019)^a^	(0.019)	(0.020)	(0.017)	(0.016)
Sec.League	0.590***	0.374*	0.335*	0.289*	0.509***
	(0.159)	(0.162)	(0.174)	(0.148)	(0.138)
Sec.League×Month	-0.010	-0.021	0.008	0.025	-0.022
	(0.024)	(0.025)	(0.026)	(0.022)	(0.021)
N	24	24	24	24	24
Pseudo *R*-squared	0.093	0.135	0.220	0.311	0.399
Alpha	0.001	0.020	0.014	0.011	0.001
*P*-value, H_0_: Alpha = 0	1.000^b^	0.066	0.169	0.103	1.000

*** *P*<0.01

* *P*<0.1

^a^ Standard error in parenthesis.

^b^ *P*-value from the likelihood-ratio test on the equality of the variances between the negative binomial and Poisson models.

This table shows that the RAE in second-tiers does not differ from that in first-tiers in any country. Moreover, we find that in all of the countries in our sample there is statistically significant evidence of RAE.

As an additional analysis, we investigate whether the RAE varies by country; we do this by inserting an interaction between country and month. No statistically significant result is found (S1 and S2 Files).

To better understand the magnitude of the overall RAE by tier, we can look at the results in terms of predicted counts by month of birth pooled through countries (see Table 4). For sake of clarity, we only report results from the Poisson regression separately for first- and second-tier leagues. These results are obtained with the command “margins” after the “poisson” output is produced. Note that since the results for the first- and second-tiers are reported separately, in each column the results are obtained from analyzing 60 observations, that is, one observation *per* month *per* country.

**Table 4:** Predicted number of players per month for first- and second-tiers separately.

	Tiers	
	First-tier	Second-tier
Variables		
Month 1	33.76	51.51
Month 2	31.51	47.91
Month 3	29.41	44.56
Month 4	27.45	41.44
Month 5	25.62	38.54
Month 6	23.91	35.85
Month 7	22.32	33.34
Month 8	20.83	31.01
Month 9	19.45	28.84
Month 10	18.15	26.83
Month 11	16.94	24.95
Month 12	15.81	23.20
N	60	60

We observe that the RAE does not change across-tiers. On average, across the five first-tier leagues, we expect 34 players to be born in the first month (i.e., January or September in England) and only about half that number to be born in the last month (i.e., December or August in England). We can compute the I_D_, expressed as the ratio between the expected number of players born in the first month and the same statistic for those players born in the last month. Therefore, for the first-tiers we obtain 33.76/15.81 = 2.13. This means that in any of the five first-tier leagues we are likely to observe that the number of players born in January is about twice the number of players born in December. On average, across the five second-tier leagues we expect 52 players to be born in the first month and about 23 to be born in the last month. Thus, in second-tiers, the I_D_ is 51.51/23.20 = 2.22, just slightly higher than the I_D_ for first-tiers.

## Discussion

This study confirms the presence of the RAE in soccer, along the lines of many other studies [4,6,21–24]. Moreover, this study contributes to the literature in two ways. First, it contributes to authors’ knowledge as no previous study has investigated whether RAE exists in second-tier leagues. This is an important research topic because lower leagues represent a second chance for players to enter first-tier competitions. In particular, players who initially struggled, who were the youngest in their age group during youth academy, may try to enter first-tier leagues or to acquire professional status by passing through teams in second-tier leagues. Second, only two previous studies in the RAE literature in sports have used count data multivariate regression models to investigate the RAE [4,14]. Our study contributes by further disseminating this methodology and by discussing how to integrate it with an interaction term to understand how the RAE varies with a certain characteristic, in our case, the term, second-tier status. Whereas Chi-square tests can only tell us that the RAE exists in both first-tier and second-tier leagues, when we apply count data multivariate regression models we additionally find that the RAE in second-tiers is the same as in the first-tiers. There is no statistically significant difference in the RAE in the two tiers. This is true for all of the top five European soccer leagues. The unequal distribution of birthdates in favor of players born early in the year is apparently as frequent in second-tier leagues as it is in first-tier leagues. An additional analysis (S1 and S2 Files) suggests that the RAE does not vary by country either. Finally, our analyses suggest that, *in this case*, it does not matter whether we use a Poisson or a negative binomial regression model. However, as good praxis, researchers should always verify whether their data are affected by overdispersion.

Although in principle, relative age should have little to no influence on the results in professional and senior matches, there are large differences in abilities and maturation during adolescence. When soccer coaches aim for match or competition victories, rather than focusing on players’ skills development, they tend to select players who have a higher probability of helping to achieve that outcome. Unfortunately, during adolescence, players are grouped by year of birth and, therefore, the same category includes players born much earlier than others. These players possess a higher level of maturity that is mistaken for higher levels of skills, thus, leading to their selection. At a professional level, soccer players differ mainly in terms of skills but in early youth categories certain physical components of the players, which are frequently highlighted (used), could be very alluring and could appear to be “skills”. Relatively younger players are often substitutes and play less than their older teammates. This situation increases their dropout chances. As previously noted, over time, this mechanism is reflected in an RAE in professional soccer, which is also apparent in second-tier leagues.

Taking the results of this research into consideration, on average, across the five first-tier leagues, we expect 34 players to be born in the first month (i.e., January or September in England), whereas we expect about half that number of players to be born in the last month (i.e., December or August in England). That is, the index of discrimination (I_D_) tells us that in any of the five first-tier leagues we are likely to observe that the number of players born in January is about twice the number of players born in December. These results do not seem to differ much in second-tier leagues. On average, across the five second-tier leagues we expect 52 players to be born in the first month and about 23 to be born in the last month. Thus, in the second-tiers, the I_D_ is just slightly higher than the I_D_ for the first-tiers. Being born in January or February does not mean that a player has better “skills”. However, it does mean that he has twice as many chances to become a professional player compared to a player born in December. Our I_D_’s are very similar to those calculated by Doyle and Bottomley [4] for the top 1,000 professionals and UEFA Under-19 Youth League players. Essentially a professional second-tier league player born in December does not have any later chance to reach the first-tier league. This is similar to Doyle and Bottomley’s [4] findings for the players they investigated, who also did not manage to reach the highest levels in soccer.

## Conclusions

This research supports previous findings of the RAE in soccer. Moreover, it shows that the RAE is the same in second-tiers as in first-tiers: *one RAE fits all tiers*, so it appears that there is no second chance for later born players to enter the first-tiers via the backdoor.

Statistically speaking, there is currently twice the chance of becoming a professional soccer player if you are born in January compared to being born in December. The RAE is a well-recorded phenomenon that should be carefully considered by coaches and clubs. The unequal distribution of players’ birthdates is not caused by differences in skill, rather, it is caused by a superficial selection process that does not take account of simple maturity differences when explaining performance gaps in youth. This superficial selection process throughout youth certainly causes talent loss that cannot be recovered later on—not even through later selection into second-tier teams. The effect is so great that even in the second-tiers the majority of players are born earlier. This reduces the chances to recover talented players who were discarded in youth simply because of lower maturity. Young players’ performance should always be evaluated by taking into account that each player’s development status might not necessarily coincide with his age. Motor-functional and soccer-specific performance assessments should also be taken into account for the purpose of talent identification. Further counter-RAE interventions might involve designing competitions without official rankings until youth athletes are older [25], ruling that at least 40% of the players should be born in the second half of the year [26] and reformulating the age-grouping rules to reduce the maximum age difference between team-mates in youth competitions (e.g., from 12 to 9–6 months [27]).

## Supporting information

S1 File Complete syntax for both Chi-square tests and regressions. (DO)

S2 File dta file. (DTA)
